# Genetic determinants of BMI, diet, and fitness interact to partially explain anthropometric obesity traits but not the metabolic consequences of obesity in men and women

**DOI:** 10.1038/s41366-026-02027-0

**Published:** 2026-02-20

**Authors:** Carmen E. Arrington, Debra K. M. Tacad, Hooman Allayee, Kristen J. Sutton, Catherine Dombroski, Nancy L. Keim, John W. Newman, Brian J. Bennett

**Affiliations:** 1 Department of Nutrition, University of California-Davis, Davis, CA USA; 2 West Coast Metabolomics Center, University of California-Davis, Davis, CA USA; 3 Department of Medicine, David Geffen School of Medicine of UCLA, Los Angeles, CA USA; 4 Department of Biomedical Informatics, University of Colorado Anschutz Medical Campus, Aurora, CO USA; 5 Department of Neurobiology, Physiology and Behavior, University of California-Davis, Davis, CA USA; 6 School of Veterinary Medicine, University of California-Davis, Davis, CA USA; 7 USDA-ARS Western Human Nutrition Research Center, Davis, CA USA

**Keywords:** Risk factors, Genetics

## Abstract

**Background:**

Understanding how genetic factors interact with diet and lifestyle to influence obesity is critical as we move towards models of precision nutrition and medicine.

**Objectives:**

To assess how genetic and lifestyle factors influence the variation in body composition and metabolic syndrome (Metsyn) risk factors.

**Methods:**

A cross-sectional sample of age/sex/BMI-balanced 18–66 year old men and women (*n* = 211) from the USDA Nutritional Phenotyping Study were included in the analysis (NCT02367287). BMI polygenic risk scores (PRS) were calculated with the pgs_calc pipeline. Associations with body composition and Metsyn traits were assessed by linear regression and ANCOVA. Explained variance was evaluated using sum of squares and partial R², with model constraint using Bayesian information criteria.

**Results:**

The PRS independently explained 15.6% of BMI variance and, after adjusting for age, sex, and genetic population structure, accounted for 11.3% of BMI variance (*p*_*ANCOVA*_ = 1.1 × 10⁻⁷). Measures of diet quality, fitness, and resting metabolic rate (RMR) showed mixed independent associations with obesity traits. In best fit models, while the PRS was significant for DXA outcomes, waist circumference, and fasting TG, the explained variance was below 3% except in android-to-gynoid ratio (3.3%), lean mass index (6.6%), and waist circumference (10.1%). The BMI PRS showed subtle associations with the metabolic/physiological consequences of obesity, only waist circumference and plasma glucose were associated with PRS. Blood pressure, triglycerides, and HDL levels were not associated with PRS for obesity.

**Conclusions:**

The genetic factors influencing BMI appear to differ from those contributing to measures of adiposity and metabolic consequences of obesity. Genetic risk of high BMI was validated in this cohort, but sex, RMR, and fitness are the more refined determinants of adiposity and dysregulated metabolism in this healthy population. Future research should be sure to utilize genetic risk predictors specifically associated with maladaptive obesity traits rather than more broad associated phenotypes.

**Clinical trial registry:**

NCT02367287

## Introduction

Obesity is a highly complex, multi-factorial disease [1]. The development and consequences of obesity are shaped by a wide range of biological, behavioral, and environmental factors. Often in large population-based studies, body mass index (BMI) is utilized to classify subjects as having obesity based on the WHO and NIH guidelines implemented in 1998, decades after BMI was validated as a disease marker by Ancel Keys in the Seven Countries Study [2, 3]. BMI has provided a standardized way to identify individuals at increased risk for chronic diseases like diabetes and heart disease and proven critical to population health. However, body size is not the only risk factor or symptom of obesity; it also includes underlying metabolic dysfunction, ectopic fat deposition, and alterations in inflammatory and hormonal signaling, all of which may precede or occur independently of elevated BMI. A holistic approach to this complexity is important in risk prediction and prevention efforts. Understanding the heterogeneity in obesity, through integrative models, will improve the effectiveness of clinical interventions and improve our understanding of underlying biological mechanisms.

Over the last two decades, the understanding of genetic contributions to obesity have expanded. While rare monogenic forms of obesity caused by single mutations in genes like leptin, MC4R, or SH2B1 can lead to severe early-onset obesity, obesity is generally a complex and polygenic phenotype [4–6]. Genome-wide association studies (GWAS) have identified numerous common variants with small individual effect sizes that collectively contribute to obesity risk, like variants in FTO and IRX3 [7, 8]. Polygenic risk scores (PRS) aggregate the effects of thousands or millions of genetic variants across the genome to provide a comprehensive risk prediction for complex obesity. While monogenic predictors are effective for identifying individuals with rare monogenic syndromes, PRS approaches are designed to assess genetic risk in the broader population. Our primary motivation was to utilize PRS to characterize genetic risk in a cross-sectional population with deep phenotyping to understand the utility of PRS in relation to other behavioral risk factors such as diet and fitness.

In this study, we evaluated the utility of a BMI-based PRS on adiposity and metabolic syndrome (Metsyn) traits in the USDA Nutritional Phenotyping Cohort. We compared the predictive performance of the PRS against other established risk factors, including age, sex, diet quality, resting metabolic rate, and fitness to better understand the genetic and non-genetic contributors to obesity as reflected by BMI in a healthy cohort.

## Methods

### Study design

A subset of participants with genetic data from the USDA Nutritional Phenotyping Study (ClinicalTrials.gov: NCT02367287), a cross-sectional study conducted at the USDA Western Human Nutrition Research Center (WHNRC) in Davis, CA, were included in this analysis (*n* = 211). A detailed description of participation and study design for the Nutritional Phenotyping Study has been previously published [9]. Briefly, generally healthy adults between the ages of 18–66 years were recruited and enrolled. Sex was used as a binary categorization and participants self-reported as either female or male during the initial screening process. Participants were excluded if they were pregnant, lactating, had undergone minor surgery recently or major surgery in the past 16 weeks, were hospitalized 4 weeks prior to their scheduled study visit, or were taking antibiotic therapy or daily medication for a diagnosed chronic disease.

Following initial screening and enrollment into the study, participants attended 2 study visits ~2 weeks apart. During the first study visit, a self-reported demographic questionnaire was given to participants and physiology measures, including body composition and fitness, were taken. On the second study visit, resting metabolic rate (RMR) and blood samples were collected following a 12-h overnight fast and after the consumption of a standardized mixed macronutrient meal challenge.

### Ethics approval and consent to participate

All methods were performed in accordance with the relevant guidelines and regulations. The study was approved by the University of California, Davis Institutional Review Board (protocol: #691654). Participants provided written informed consent and received monetary compensation for participation in this study.

### Body composition

Body weight and height were measured using a calibrated scale and wall-mounted stadiometer, respectively. Body fat and lean mass were measured using dual x-ray absorptiometry (DXA, Hologic Discovery QDR Series with Apex 13.3.7, Hologic, MA, USA) by a trained and licensed technician. Pre-menopausal female participants reported the date of their last menstrual period and completed a spot urine pregnancy test prior to scan. Whole body scans were performed on participants to provide data on total lean body mass and total fat mass. Body fat percentage (BF%) was an output from the DXA scan, reflecting the total fat mass divided by the total body mass. Lean mass index (LMI) was calculated as total lean body mass (kg) divided by squared height (m^2^). The “trunk” is the area starting from the bottom of the chin to the pelvis, excluding the arms and legs. Trunk fat percentage (TF%) was an output from the DXA scan. The trunk area consists of the android and the gynoid region. Android fat mass is fat accumulation in the area 20% of the distance from the crest to the chin, excluding the extremities, while gynoid fat mass is fat accumulation in the area around the hips/pelvis. The android-to-gynoid fat mass ratio (AGR) was used to infer the distribution of fat mass and calculated as the total android fat mass divided by the total gynoid fat mass. In the manuscript, we use the term adiposity to refer to body fat measured by DXA, including regional fat distribution.

### Metabolic syndrome traits

The five classical Metsyn traits – waist circumference, fasting triglycerides (TG), fasting high density lipoprotein cholesterol (HDL-c), fasting glucose, and blood pressure (systolic (sBP) and diastolic (dBP)) – were collected in the study. Waist circumference was measured at the smallest horizontal circumference between the ribs and iliac crest. Fasting plasma TG, HDL-c, and glucose were measured on a Cobas Integra 400 Plus (Roche Diagnostics Corporation). Seated blood pressure measurements were obtained using the CARESCAPE V100 Vital Signs Monitor (GE Healthcare) following a 5 to 10-minute rest.

### Dietary assessment

Dietary recalls were obtained using the Automated Self-Administered 24-hour (ASA24) dietary recall tool from the National Cancer Institute of the National Institutes of Health [10]. A detailed description of the dietary data collection and processing has been previously published [11]. Briefly, participants were familiarized with the ASA24 dietary recall process by trained personnel and were guided on how to report food intake online. In the 10–14 days between study visits, participants were prompted to complete 3 unscheduled recalls at home, which were split between 2 weekdays and 1 weekend day to account for variations in food intake throughout the week. Dietary quality for 3-day food intake averages was assessed using the Health Eating Index (HEI), which estimates adherence to the US Dietary Guidelines for Americans [12]. The total HEI score was used for estimating dietary quality in analyses.

### Resting metabolic rate (RMR)

Prior to the second study visit, participants consumed a standardized dinner meal and ceased any food or beverage consumption (except for water) 12 h before their scheduled visit to the center. Upon arrival, a trained physiologist measured RMR through indirect calorimetry by a metabolic cart (TrueOne 2400, ParvoMedics, Sandy, UT). Participants laid semi-reclined and were undisturbed for 5 min prior to testing. Respiratory gases were then collected for ~15–20 min to measure oxygen consumption and carbon dioxide production. These data were used in the Weir equation (without urinary nitrogen) to estimate RMR [13]. RMR was subsequently divided by body mass to account for innate differences in RMR due to body size.

### Fitness

The YMCA 3-minute step test is a validated exercise test to evaluate participant cardiorespiratory fitness [14, 15]. Prior to the fitness assessment, the Physical Activity Readiness Questionnaire (PAR-Q, American College of Sports Medicine, Indianapolis, IN) was administered to determine if the participant could complete the exercise test safely [16]. Participants who answered “yes” to any of the questions in the readiness questionnaire related to heart, bone, or joint conditions, or recent injuries were categorized as “Unknown” for fitness level. For the fitness assessment, a heart rate monitor (Polar Watch V800, Polar Electro, Kemplele, Finland) was secured to the wrist and resting heart rate was collected. Participants stepped up on a 12-inch box, one foot at a time, and then stepped down, one foot at a time, for 3 min. A metronome set to 96 beats per minute was used to indicate when participants should step with the alternating foot. Seated heart rate was measured during the first minute of the recovery period. The post-exercise heart rate was stratified using participant age and sex to categorize them into “Very Poor,” “Poor,” “Below Average,” “Average,” “Above Average,” “Good,” or “Excellent” fitness levels.

### Genotype data

Participants consented to genotyping and samples were prepared using PAXgene Blood DNA kits (Qiagen, Germantown, MD) for sequencing, which has been described previously [17, 18]. DNA was extracted and purified according to the manufacturer’s instructions and sent to UCLA Neuroscience Genomics Core for genotyping. The Infinium Global Screening Array version 3 (Illumina Infinium GSA-24 v3.0 BeadChip) was used to type 654,027 high-quality SNPs across the human genome. Data underwent quality control measures including excluding duplicate single nucleotide polymorphisms (SNPs), low quality (<95%) genotyping and missing SNPs (>1%), SNPs that fail Hardy-Weinberg Equilibrium (HWE < 0.0001), monomorphic SNPs, SNPs with an indeterminate allele, and SNPs that map to multiple probes. Related participants with an identity by state value greater than 0.2 were identified, and inclusion of relatives was evaluated for impact on downstream analysis. Genetic data from the sample was subsequently imputed using the TOPMed imputation server to impute the genotype array data. The server facilitates a multi-step process to enhance genotype accuracy including quality control to filter out low-confidence genotypes. Briefly, the genetic data, originally in Hg19, underwent LiftOver followed by quality control, which returned a reference overlap of 93.79% (chr 1–10), 93.99% (chr 11–22), and 92% (chr X) with the TOPMed R3 Hg38 reference panel. Principal component (PC) analysis was used to generate PCs which account for overall genomic variation and population structure. The first 5 PCs were utilized in analyses and describe >50% of the genetic population structure.

### PRS calculation

Polygenic risk scores describe the combined risk of several risk-inducing SNPs previously identified in GWAS. The PRS for BMI was calculated using the PGS Catalog and pgs_calc pipeline [19] made accessible by the nf-core community [20]. Briefly, the harmonized GRCh38 PGS002313 score file, from Weissbrod et al, was downloaded from PGS Catalog and subsequently used in pgs_calc along with the study sample’s imputed genetic data [21]. This score was based on the cumulative effect of more than 1 million genetic variants identified through large-scale GWAS in the UK Biobank. The pgs_calc pipeline matches variants in the scoring files against variants in the target dataset and calculates PRS for all samples (using linear sum of weights and dosages). The target dataset (USDA Nutritional Phenotyping Cohort) was an independent sample from the discovery GWAS (UK Biobank). Genetic variant coverage was high for this PRS (96.1%). The calculated PRS was then normalized and centered around 0 prior to inclusion in the analysis models (Supplementary Fig. 1). PRS quintiles were utilized only in data visualization to compare the BMI between low, medium, and high-risk groups as the genetic effects were more likely to manifest in the extremes; PRS quintile 1 was assigned “low-risk”, PRS quintile 2, 3, and 4 were assigned “mid-risk”, and PRS quintile 5 was assigned “high-risk.” The continuous, normalized PRS variable was utilized in the subsequent linear regression models.

The PRS was validated using a larger sample set of the USDA cohort (*n* = 230) of participants with age, sex, BMI, and genetic data. BMI was used to classify participants with obesity (BMI > 30) and subsequently used to find the discriminate ability of PRS to identify participants with obesity using area under the receiver operating curve (AUROC). A linear regression model was used to evaluate the association and R^2^ of PRS predicting BMI after adjusting for age, sex, and principal components.

### Independent associations with DXA phenotypes

**Fig. 1:**
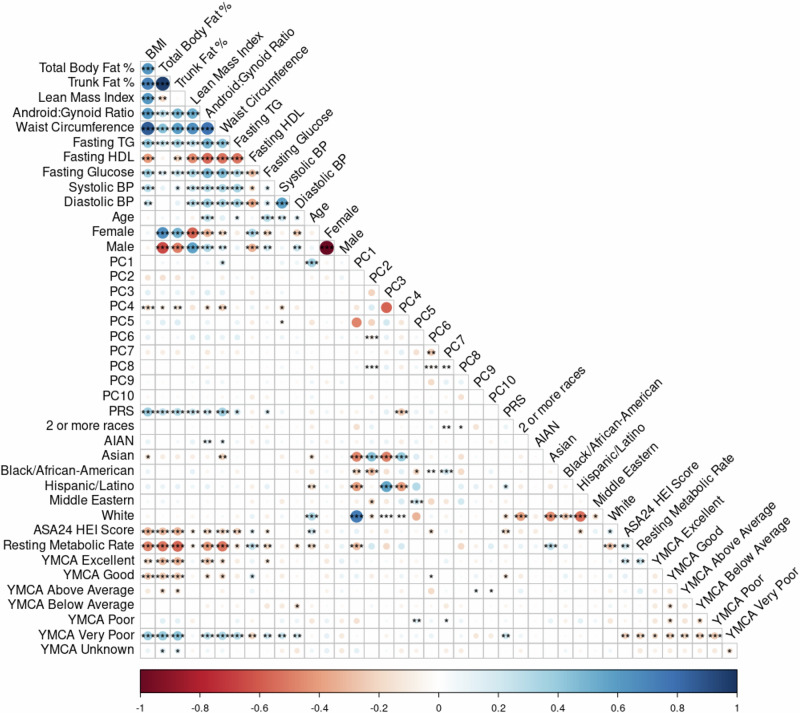
Spearman correlation plot. The plot of the Spearman correlations including all predictor and response variables examined in this sample (*n* = 211). Associations between variables are colored according to the sliding gradient from red (−1) to blue (1) and significant associations are denoted by asterisk (*<0.5, **<0.01, ***<0.001). Among the covariates, fitness shows a moderate correlation with the DXA phenotypes, as does HEI, indicating some association between fitness level and dietary quality with body composition. PRS is correlated with DXA phenotypes, supporting the role of genetic factors in body composition variation. Notably, the first 5 principal components (PCs 1–5) describing the genomic variation and population structure (cumulative eigenvalue = 20.8) show distinct correlations with the reported DXA phenotypes, age, PRS, and other variables, while PCs 6–10 are largely redundant (PC 1–10 cumulative eigenvalue = 28.5). This redundancy suggests that only the first five PCs are of primary interest in subsequent analyses, as they capture the most meaningful variation across the phenotypes. Additionally, these PCs exhibit significant correlations with the participant’s self-reported race.

Spearman correlations were used to assess monotonic relationships between selected variables and covariates (Fig. 1). The critical Spearman’s rho value for statistical significance was approximated based on a sample size of 211 and a significance level of 0.05.

Linear regression models including all standardized predictor variables were used to assess percent variation using sum of squares to describe the independent contribution of each predictor to each outcome. Variance inflation factor was used to assess multicollinearity.

### Full model building

PRS, HEI, RMR, and fitness variables were evaluated along with age, sex, and genetic PC 1–5 in a full model. First, variables were standardized, mean centered with an SD of 1, to ensure comparability in combined models by making each variable more consistent and easier to understand. Next, variables were examined using sum of squares and partial R^2^. The sum of square criteria measures the contribution of each predictor, without accounting for the influence of other variables, while the partial R^2^ criteria measures the unique contribution of each predictor accounting for the remaining variables. Models for each phenotype were subsequently evaluated using stepwise, bi-directional regression analysis and evaluated using Bayesian information criteria (BIC) to determine the most explanatory model. Each regression model was iteratively built by adding and removing variables, alternating between forward and backward selection at each step, using BIC to determine which variables to keep in the model at each iteration using the MASS package in R [22].

### Statistical methods and computing

R (Version 4.4.1, R Foundation for Statistical Computing; Vienna, Austria) [23] was used for statistical analysis and visualizations. Distribution transformations for PRS were described above. DXA phenotypes and covariates were normalized, if necessary, using logarithmic transformations; BMI, LMI, waist circumference, fasting TG, and fasting HDL-c were normalized, distribution histograms are in Supplementary Fig. 2. A significance level of α = 0.05 was used throughout the manuscript. This research was supported by the Ceres High-Performance Cluster computing network hosted by USDA-ARS.

## Results

### Participant demographics/study design

In this cohort, 211 participants had complete data for subsequent analyses (Supplementary Fig. 3). Among these participants, there were 104 females (49%) with a median age of 43 y (IQR, 18–66 y). Of the 211 participants, 140 (66%) were White, 27 (13%) were Hispanic/Latino, 19 (9%) were Asian, and 14 (7%) reported as being of 2 or more ethnicities (Table 1). There are significant differences between PRS groups by BMI and DXA phenotypes, but not in age, race, sex, diet quality, RMR, or fitness. Identity by state identified 12 pairs of first-degree relatives in our sample.

**Table 1:** Participant characteristics across PRS groups^a^.

Characteristic	Units	PRS groups			*P*-value^b^
		Low-risk (*n* = 43)	Mid-risk (*n* = 126)]	High-risk (*n* = 42)	
PRS002313, mean [range]		−1.5 [−3.6, −0.9]	0 [−0.9, 0.8]	1.3 [0.8, 2.5]	<2.2 × 10^−16^
Age, mean [range]	y	40.9 [19–65]	38.7 [19–65]	38.8 [19–62]	0.54
Self-reported Race, *n* (%)					0.55
American Indian or Alaska Native		0 (0)	1 (0.8)	0 (0)	
Asian		4 (9.3)	11 (8.7)	4 (9.5)	
Black/African-American		0 (0)	5 (4.0)	1 (2.4)	
Hispanic/Latino		3 (7.0)	15 (11.9)	9 (21.4)	
Middle Eastern		2 (4.7)	1 (0.8)	0 (0)	
White		32 (74.4)	84 (66.7)	24 (57.1)	
2 or more races		2 (4.7)	8 (6.3)	4 (9.5)	
Not reported		0 (0)	1 (0.8)	0 (0)	
Sex, n (%)					0.54
Female		18 (41.3)	64 (50.8)	22 (52.4)	
Male		25 (58.1)	62 (49.2)	20 (47.6)	
BMI, mean [range]	kg/m^2^	24.9 [20.1–34.5]	26.8 [17.9–42.9]	30.3 [21.7–43.3]	2.8 × 10^−6^
HEI score, mean [range]		63.9 [41.4–86.8]	60.8 [32.1–91]	57.4 [22.5–83.2]	0.20
Android-to-Gynoid Fat Ratio, mean [range]		0.42 [0.19–0.79]	0.43 [0.18–1.03]	0.52 [0.21–0.89]	0.0036
Total Body Fat, mean [range]	%	25.6 [10.4–46.5]	27.1 [9.8–52.2]	33.1 [15.5–46.9]	0.0020
Lean Mass Index, mean [range]	kg/m^2^	16.8 [12.9–20.2]	17.6 [13.2–25.0]	18.5 [14.1–23.5]	0.015
Trunk fat, mean [range]	%	25.6 [8.3–46.9]	27.2 [9.8–51.9]	34.4 [14.8–51.3]	3.5 × 10^−4^
Waist circumference	cm	81.0 [66.7–109.2]	84.5 [63.3–127.6]	94.3 [64.3–138.4]	2.9 × 10^−6^
Fasting triglycerides	mmol/L	0.92 [0.44–2.58]	1.01 [0.40–3.07]	1.11 [0.38–2.41]	0.044
Fasting HDL-c	mmol/L	1.39 [0.83–2.10]	1.35 [0.72–2.42]	1.33 [0.71–2.5]	0.77
Fasting glucose	mmol/L	5.09 [4.38–6.26]	5.21 [3.46–7.35]	5.36 [4.65–7.74]	0.069
Systolic blood pressure	mmHg	118.9 [95–139]	118.7 [92–139]	121.7 [102–139]	0.31
Diastolic blood pressure	mmHg	68.8 [51–94]	68.0 [52–90]	70.0 [50–90]	0.39
YMCA step test, *n* [%]					0.58
Excellent		3 [7.0]	11 [8.7]	3 [7.1]	
Good		8 [18.6]	15 [11.9]	4 [9.5]	
Above average		4 [9.3]	13 [10.3]	3 [7.1]	
Average		6 [14.0]	16 [12.7]	2 [4.8]	
Below average		7 [16.3]	18 [14.3]	3 [7.1]	
Poor		6 [14.0]	18 [14.3]	7 [16.7]	
Very poor		8 [18.6]	26 [20.6]	16 [38.1]	
Not reported		1 [2.3]	9 [7.1]	4 [9.5]	
RMR, mean [range]	kcal/day/kg	21.5 [13.5–27.6]	21.3 [13–30.9]	20.5 [13.1–28.8]	0.22

^a^Means reported as geometric means of continuous variables in all cases except PRS002313 due to negative values.

^b^*P*-values calculated using Kruskal–Wallis test for continuous variables and Pearson χ2 test for categorical variables.

### Polygenic risk score (PRS) for obesity in nutritional phenotyping cohort

**Fig. 2:**
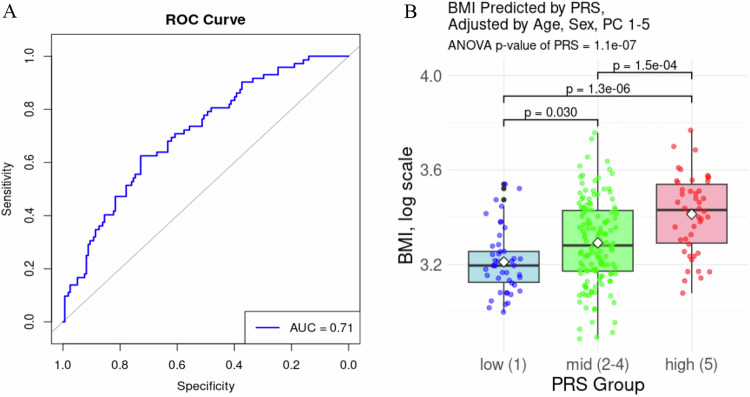
PRS validation. The receiver operating characteristic (ROC) curve (**A**) evaluated the performance of the polygenic score (PRS, *PGS002313*) in predicting BMI classification (disease: BMI > 30 vs. non-disease). The area under the curve (AUC) is 0.71, providing a measure of the model’s discriminative ability, with higher values indicating better prediction. A diagonal reference line (AUC = 0.5) is shown, representing random classification. **B** The boxplot visualizes the relationship between BMI (log-transformed) and PRS groups, adjusted for age, sex, and the first 5 genetic principal components (PCs 1–5). The plot includes jittered individual data points, mean values for each PRS group, and pairwise comparisons between groups with corresponding FDR-corrected p-values indicated above the plot.

The linear regression model to validate the PRS in this cohort explained 15.6% of the variance in BMI, suggesting a moderate relationship between the genetic risk score and BMI. A binary variable was created to classify individuals with obesity (BMI > 30 kg/m^2^, *n* = 72). The area under the receiver operating characteristic curve (AUC) for the PRS to predict obesity was 0.71, indicating that the PRS demonstrates good discriminatory ability (Fig. 2A). These metrics suggest that the PRS provides predictive value for both BMI and obesity, as defined by BMI > 30, in our sample. Most PRS models in the PGS Catalog show AUCs between 0.6 and 0.7, the score of 0.71 demonstrates a moderately strong, independent predictive performance prior to the inclusion of other clinical and demographic risk factors [24].

A Spearman correlation plot of all variables included in the models for all adiposity and metabolic traits (Fig. 1) highlights several key relationships between predictors and outcomes. When further adjusted for age, sex, and the first 5 genetic PCs, the ANCOVA test indicated that the PRS remained a significant predictor of BMI (*p*_ANCOVA_ = 1.1 × 10^−7^) and explained 11.3% of the variation in BMI. While variance in BMI differed significantly between PRS groups (Levene’s test *p* = 0.015), the PRS effect remained highly significant when accounting for heterogeneity (Welch’s ANOVA F = 21.0, *p* = 2.5 × 10^−8^). Figure 2B visualizes the boxplot of BMI by PRS group and demonstrates that individuals in the high-risk group had higher BMI, with statistically significant differences between the groups.

### Polygenic risk score and obesity traits

The comprehensive phenotyping protocol and recruiting strategy of the Nutritional Phenotyping Study provides an opportunity to understand the contribution of commonly used PRS to more precise measures of adiposity and Metsyn. Higher PRS is significantly associated with higher adiposity and waist circumference, but not other metabolic traits (Table 2). While there is a significant association, the contribution of PRS to these traits is low, except for lean mass index and waist circumference (Supplementary Table 1).

**Table 2:** Independent associations of BMI PRS with adiposity and metabolic syndrome outcomes.

Trait	ANOVA *P*-value	PRS Group Comparison	Estimate	SE	Pairwise *P*-value
BMI, log scale	1.2 × 10^−6^	low (1) - mid (2–4)	−0.0678	0.031	0.072
		low (1) - high (5)	−0.1924	0.038	7.6 × 10^−4^
		mid (2–4) - high (5)	−0.1246	0.030	2.6 × 10^−6^
Body Fat Percentage	1.4 × 10^−4^	low (1) - mid (2–4)	−0.634	1.314	0.88
		low (1) - high (5)	−5.527	1.624	3.5 × 10^−3^
		mid (2–4) - high (5)	−4.893	1.300	1.9 × 10^−3^
LMI, log scale	2.8 × 10^−5^	low (1) - mid (2–4)	−0.0596	0.020	0.012
		low (1) - high (5)	−0.1153	0.025	4.2 × 10^−5^
		mid (2–4) - high (5)	−0.056	0.020	0.014
Trunk Fat Percentage	8.9 × 10^−5^	low (1) - mid (2–4)	−1.222	1.600	0.73
		low (1) - high (5)	−7.161	1.978	1.6 × 10^−3^
		mid (2–4) - high (5)	−5.940	1.584	1.6 × 10^−3^
Android to Gynoid Ratio	1.3 × 10^−4^	low (1) - mid (2–4)	−0.035	0.026	0.36
		low (1) - high (5)	−0.121	0.032	1.8 × 10^−3^
		mid (2–4) - high (5)	−0.085	0.026	4.4 × 10^−3^
Waist Circumference, log scale	7.6 × 10^−8^	low (1) - mid (2–4)	−0.053	0.024	0.073
		low (1) - high (5)	−0.164	0.030	9.5 × 10^−7^
		mid (2–4) - high (5)	−0.111	0.024	2.5 × 10^−5^
Fasting Triglycerides, log scale	0.077	low (1) - mid (2–4)	−0.099	0.078	0.49
		low (1) - high (5)	−0.188	0.097	0.39
		mid (2–4) - high (5)	−0.088	0.077	0.49
Fasting HDL-c, log scale	0.57	low (1) - mid (2–4)	0.047	0.048	0.89
		low (1) - high (5)	0.059	0.060	0.89
		mid (2–4) - high (5)	0.012	0.048	0.97
Fasting Glucose	8.2 × 10^−3^	low (1) - mid (2–4)	−0.174	0.086	0.17
		low (1) - high (5)	−0.327	0.106	0.020
		mid (2–4) - high (5)	−0.154	0.085	0.17
Systolic BP	0.24	low (1) - mid (2–4)	0.864	1.825	0.88
		low (1) - high (5)	−1.681	2.255	0.88
		mid (2–4) - high (5)	−2.545	1.805	0.88
Diastolic BP	0.34	low (1) - mid (2–4)	0.869	1.583	0.85
		low (1) - high (5)	−1.200	1.956	0.85
		mid (2–4) - high (5)	−2.069	1.566	0.85

Linear regression models examined the association of the polygenic risk score of BMI with adiposity and metabolic syndrome traits, adjusted for age, sex, and principal components 1–5. The ANOVA *p*-value for each model is listed as well as each pairwise comparison between the three risk groups by PRS (low, mid, high). The effect size, standard error, and FDR-corrected pairwise *p*-value are listed.

### Independent associations of diet quality, resting metabolic rate, and fitness with adiposity

Additional factors which were expected to contribute to variation in body composition and obesity traits include diet quality, resting metabolic rate, and fitness. The independent contribution of each of these additional factors to specific outcomes varies. For example, diet quality accounts for ~5–10% of variance in 5 of the outcomes with the largest contribution (10.4%) to TF% indicating an association between regional accumulation of adiposity and diet quality but otherwise little evidence for an association with the metabolic consequences of obesity. RMR and fitness contributed proportionally more to obesity traits than genetic risk and diet quality. RMR accounted for 10–20% of variance in DXA outcomes, except LMI, and waist circumference. Fitness accounted for 5–10% of the variance across all outcomes, except LMI (Supplementary Table 1). Additional ROCs were tested to compare other predictive models including a covariate only model, a simple PRS model with age, sex, and PCs, and the full model including all predictor variables (Supplementary Fig. 4). Comparing these models, we find all models are significantly different from each other, and specifically the full model, including PRS, significantly improved the predictive capability of the covariate only model using DeLong’s test for two correlated ROCs (*p* = 0.03).

### Best fit models

Evidence of independent associations of diet quality, RMR, and fitness highlighted the potential contributions of each variable to adiposity; however combined effects would better estimate their contribution to obesity. The relationships between covariates and the potential for multicollinearity were assessed to ensure the stability and interpretability of the results. The Spearman correlation identified several correlations between predictor variables; however, the calculated variance inflation factor (VIF) values did not indicate evidence of multicollinearity (VIF < 2).

We examined the possibility of an interaction between PRS and the other predictors in a logistic regression model of obesity using likelihood ratio tests. Evidence of predictor interactions were not detected (*p* > 0.05).

**Fig. 3:**
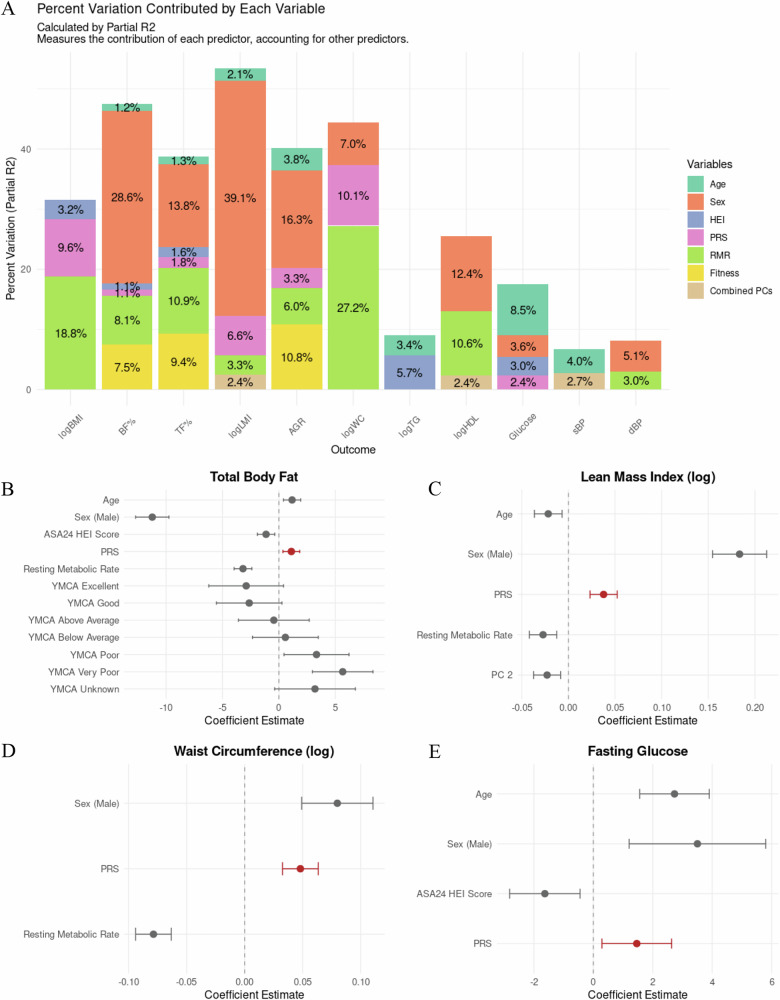
Best fit model partial R^2^ and regression results. **A** Percent variation contributed by each variable. Stacked bar chart showing the relative contribution of predictor variables to model variance (calculated by partial R²) for each outcome. Only predictor variables retained by best-fit models using stepwise bidirectional selection with BIC are shown. Variables include: Age; Sex; ASA24 HEI Score; Polygenic Score (PRS); Resting Metabolic Rate (RMR); Fitness measures; Combined Principal Components (PCs). Each bar represents the total explained variance, with segments proportional to each variable’s unique contribution after accounting for other predictors. **B**–**E** Regression coefficient estimates with 95% confidence intervals. Forest plots displaying standardized coefficient estimates and confidence intervals for significant predictors of: **B** Total body fat percentage, **C** Lean mass index (log-transformed), **D** Waist circumference (log-transformed), and **E** Fasting glucose. Points represent standardized coefficient estimates; horizontal lines indicate 95% confidence intervals. PRS coefficients are highlighted in red for easy identification among predictors. Dashed vertical line at zero indicates null effect. Variables shown are those with non-zero coefficients in the final models.

To assess the contribution of several variables on adiposity we used BIC to identify an overall model of adiposity. The most-stringent, best fit model for BMI included only HEI, PRS, and RMR, which was fit using stepwise, bidirectional model building. BF% was best modeled by age, sex, HEI, PRS, RMR, and fitness (Fig. 3, Supplementary Table 1). For the remaining DXA phenotypes, RMR and PRS were uniformly retained, but HEI was excluded in the model of LMI, and fitness was excluded in the model of LMI and AGR. Figure 3 summarizes the best fit models for all outcomes using partial R^2^ as well as the standardized regression results of a subset of the outcome models which retained PRS.

We identified the unique proportion of variance each variable contributed to the best fit model of each adiposity outcome, as quantified by partial R² values. In Supplementary Table 1, the proportion of variance by each variable using sum of squares (SS) and partial R² is compared. Only the variables in the best fit model have partial R² values to reflect the dependent nature of partial R^2^ values. Additionally, Supplementary Table 1 lists the standardized beta coefficients and standard errors of each variable included in the best fit model for each outcome. In combination with the partial R^2^, the standardized coefficients quantify the strength and directional effect of each variable on the outcome.

## Discussion

This study sought to assess the genetic risk prediction of high BMI in a healthy cohort and to assess how this genetic risk related to obesity traits like adiposity and metabolic dysregulation. Additionally, we sought a holistic view of the complex etiology of obesity and further characterized the contribution of diet quality and fitness to measures of adiposity. Specifically, we acknowledged the roles of age, sex, genetics, diet quality, RMR, and fitness in contributing to body composition and Metsyn traits. We evaluated the effectiveness of the BMI-based PRS to predict obesity-related traits including adiposity and Metsyn. Our findings suggest that a more nuanced understanding of current genetic risk could better define the complex association between adiposity and metabolic disease phenotypes. Both an improved understanding of an individual’s genetic risk of obesity as well as how specific modifiable risk factors contribute to obesity regardless of genetic risk are critical as we begin to develop models of precision health.

While the PRS was validated as a predictor of BMI in this cohort, the PRS was only comparably predictive of LMI and waist circumference, not adiposity nor Metsyn traits. This suggests that the PRS identified the genetic determinants to accumulate lean mass and/or body size, not specifically adiposity or the metabolic consequences of obesity. This identifies a key limitation of using BMI to generate PRS in large-scale genomic risk studies investigating the health consequences of obesity [25, 26]. Developing and utilizing PRS that better reflect the metabolic dysregulation leading to, and associated with, increased adiposity will improve both obesity prevention and treatment. This approach will be important as we move from large-scale genomic studies towards models that predict individual responses to specific medications, diets, or other intervention strategies.

The contribution of sex to the explained variance of body fat accumulation and waist circumference is in stark contrast to that of BMI. Typically, PRS are calculated from GWAS summary statistics in both male and female participants, ignoring sex differences, with the underlying assumption that the effect size of risk alleles would be the same in both males and females. This is likely why there is a minimal contribution of sex to the variance captured for BMI, as it was adjusted for. However, we saw that sex as a predictor did not capture the same variance in BF%. Sex-specific PRS have been discussed as a method to capture risk for sex differences in certain traits and diseases. Specifically, comparisons of sex-agnostic and sex-specific PRS found differences in traits related to the waist-to-hip ratio [27]. Additionally, it is possible that the inclusion of sex in the model is accounting for some genetic differences that could otherwise be attributed to a more specific body composition PRS. Overall, these data reinforce the notion that sex is critically important and ascertaining genetic risk using sex-stratified analysis is likely to refine our understanding of complex diseases, like obesity.

There are several limitations to this study. While the study population was ethnically representative of the greater Californian population [28], the recruitment strategy drove a healthy volunteer bias. Diet quality contributed minimally to the overall explained variance (<4%) which may be due to the relative health of the participants and/or reflect the broad nature of the HEI that aggregates various dietary factors. We do note that the average HEI in this sample was slightly higher than reported in the general population of the United States [29]. The original PRS by Weissbrod et al. [21] was trained using individuals of European genetic ancestry, and only 61% of the current sample self-identified as White. Such European-derived PRSs are known to be less accurate when applied to other genetic ancestry groups [30, 31], and, while we did not identify any differences in PRS group by self-reported race, there are differences between genetic ancestry clusters. Additionally, the use of YMCA fitness scores of cardiovascular fitness may not fully capture the complexity of physical fitness, particularly with respect to muscle mass or strength. Further research should explore the long-term effects of dietary interventions on fat distribution in genetically predisposed individuals. Additionally, while diet quality and fitness have been identified as modifiable factors in body composition and obesity, the combined contribution of <10% to BF% suggests that future studies should also seek to include additional predictors from data sources providing broad domain coverage such as microbiome and/or the metabolome. Lastly, it is likely that there were genetic correlations between BMI, adiposity traits, and metabolic traits, due to overlapping genetic architecture [32–35], which were unaccounted for; however, due to the minimal contribution of PRS across traits we did not believe this to be a necessary adjustment to make.

In conclusion, our study demonstrates that genetic predisposition, lifestyle factors such as fitness, and diet quality independently contribute to adiposity. The broad use of BMI may not describe equivalent proportions of genetic risk of more precise body composition measures. More importantly, the genetic risk of obesity, as defined by BMI, was not significantly associated with the metabolic consequences of obesity reinforcing again the overall complexity between obesity and the risk of other chronic diseases. As fitness and RMR play critical roles in adiposity and metabolic risk, interventions targeting fitness and diet quality could offer effective strategies for improving body composition and reducing the risk of obesity-related diseases.

## Supplementary information

Supplementary Material

## Data Availability

Data described in the manuscript will be made available upon request.
