# Supplementary material for: Genetic determinants of BMI, diet, and fitness interact to partially explain anthropometric obesity traits but not the metabolic consequences of obesity in men and women
Source: Int J Obes (Lond). 2026 Feb 20;50(4):938–46. doi: 10.1038/s41366-026-02027-0 (PMC13056523; doi:10.1038/s41366-026-02027-0)
Supplement: Supplementary file 1 — Supplementary Material [file 41366_2026_2027_MOESM1_ESM.docx]

**Title:** Genetic determinants of BMI, diet, and fitness interact to partially explain anthropometric obesity traits but not the metabolic consequences of obesity in men and women

**Authors:** Carmen E. Arrington^1^, Debra K.M. Tacad^2^, Hooman Allayee^3^, Kristen J. Sutton^4^, Catherine Dombroski^5,6^, Nancy L. Keim^1^, John W. Newman^2,6^, Brian J. Bennett^1,6^

**Online Supplementary Material**

*
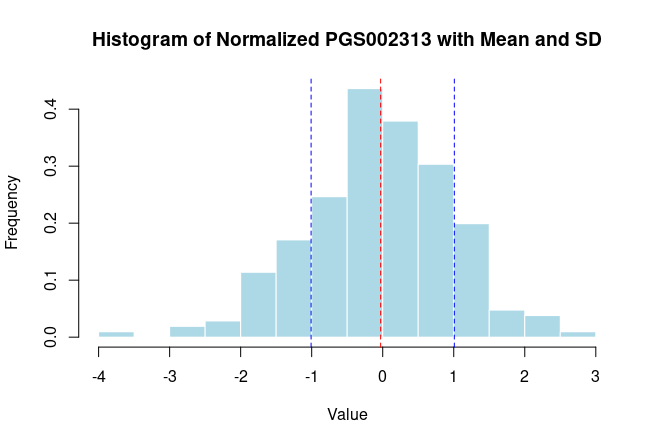
*

**Supplementary Figure 1.** The distribution of the normalized polygenic risk score (PRS) for BMI developed from the PGS Catalog. The mean (red dashed line) and SD (blue dotted line) of the sample are depicted on the plot.


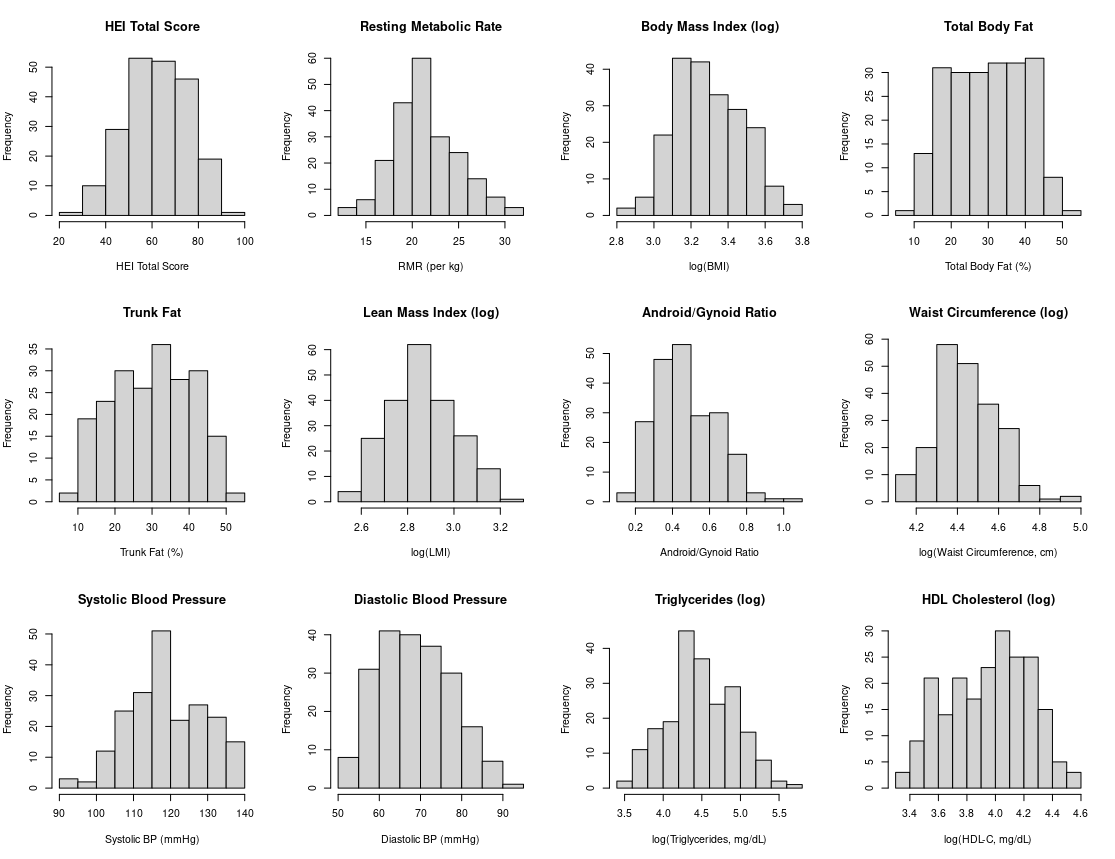


**Supplementary Figure 2.** Assessment of data normality and log transformation effects for continuous variables. Normality was assessed for 13 continuous variables using Shapiro-Wilk tests before and after natural log transformation as well as visual inspection. Log transformation was recommended and applied to 5 variables: BMI, lean mass index (LMI), waist circumference, triglycerides, and HDL cholesterol, based on substantial improvements in normality metrics. Histograms show distributions after transformation (log scale) where applied.

Participants fully complete in study (n=358)

Participants with genetic data (n=237)

Missing genetic data (n=121)

Missing diet quality data (n=6)

Missing lean mass data (n=1)

Participants with genetic data and DXA adiposity outcomes (n=230)

Sample used for PRS validation

Missing waist circumference (n=4)

Missing blood pressure (n=10)

Outlier Data:

Fasting TG > 350 mg/dL (n=2)

Fasting HDL-c > 100 mg/dL (n=2)

Fasting Glucose > 300 mg/dL (n=1)

Participants with data available (n=211)

**Supplementary Figure 3.** Flow chart showing availability of data throughout the analysis.

1. Withdrawal (n=7)
2. Exclusions (n=20)
3. Scheduling conflicts (n=8)

Participants between 18-66 years with BMIs between 18.5-45 kg/m2 enrolled in Nutritional Phenotyping Study (n=393)

**Supplementary Table 1.** Summary of Results of Obesity-related Traits in Model Building Process: Sum of Squares, Partial R² Contributions, and Regression Results.

| Outcome | Variables | Sum of Squares (%) | Partial R² (%) | Beta Coefficient | Standard Error | P-Value |
| --- | --- | --- | --- | --- | --- | --- |
| BMI, log scale | Age | 0.1 |  |  |  |  |
|  | Sex | 0.3 |  |  |  |  |
|  | HEI | 11.0 | 3.2 | -0.03 | 0.01 | 1.0×10^-3^ |
|  | PRS | 10.5 | 9.6 | 0.06 | 0.01 | 2.8×10^-8^ |
|  | RMR | 18.6 | 18.8 | -0.08 | 0.01 | 5.8×10^-14^ |
|  | Fitness | 6.9 |  |  |  |  |
|  | Combined PCs | 3.1 |  |  |  |  |
| Body Fat Percentage | Age | 0.5 | 1.2 | 1.17 | 0.39 | 2.9×10^-3^ |
|  | Sex, Male | 43.2 | 28.6 | -11.22 | 0.75 | 2.9×10^-34^ |
|  | HEI | 7.4 | 1.1 | -1.14 | 0.39 | 4.2×10^-3^ |
|  | PRS | 3.0 | 1.1 | 1.10 | 0.38 | 4.1×10^-3^ |
|  | RMR | 13.1 | 8.1 | -3.18 | 0.40 | 1.4×10^-13^ |
|  | Fitness | 7.5 | 7.5 |  |  |  |
|  | Excellent |  |  | -2.90 | 1.69 | 0.088 |
|  | Good |  |  | -2.63 | 1.48 | 0.077 |
|  | Above Average |  |  | -0.45 | 1.60 | 0.78 |
|  | Below Average |  |  | 0.57 | 1.49 | 0.70 |
|  | Poor |  |  | 3.33 | 1.47 | 0.024 |
|  | Very Poor |  |  | 5.66 | 1.37 | 5.2×10^-5^ |
|  | Unknown |  |  | 3.20 | 1.82 | 0.081 |
|  | Combined PCs | 1.4 |  |  |  |  |
| Trunk Fat Percentage | Age | 0.8 | 1.3 | 1.32 | 0.47 | 5.1×10^-3^ |
|  | Sex, Male | 25.2 | 13.8 | -8.29 | 0.90 | 5.6×10^-17^ |
|  | HEI | 10.4 | 1.6 | -1.47 | 0.47 | 2.2×10^-3^ |
|  | PRS | 4.4 | 1.8 | 1.52 | 0.46 | 1.1×10^-3^ |
|  | RMR | 17.5 | 10.9 | -3.93 | 0.48 | 3.3×10^-14^ |
|  | Fitness | 9.4 | 9.4 |  |  |  |
|  | Excellent |  |  | -4.48 | 2.03 | 0.028 |
|  | Good |  |  | -3.61 | 1.78 | 0.044 |
|  | Above Average |  |  | -0.47 | 1.92 | 0.81 |
|  | Below Average |  |  | 0.43 | 1.79 | 0.81 |
|  | Poor |  |  | 3.60 | 1.77 | 0.043 |
|  | Very Poor |  |  | 6.21 | 1.64 | 2.1×10^-4^ |
|  | Unknown |  |  | 3.38 | 2.19 | 0.12 |
|  | Combined PCs | 1.6 |  |  |  |  |
| LMI, log scale | Age | 1.1 | 2.1 | -0.022 | 0.01 | 4.8×10^-3^ |
|  | Sex, Male | 34.0 | 39.1 | 0.184 | 0.02 | 1.0×10^-26^ |
|  | HEI | 2.0 |  |  |  |  |
|  | PRS | 6.0 | 6.6 | 0.038 | 0.01 | 8.3×10^-7^ |
|  | RMR | 2.8 | 3.3 | -0.027 | 0.01 | 3.8×10^-4^ |
|  | Fitness | 1.5 |  |  |  |  |
|  | Combined PCs | 3.7 |  |  |  |  |
|  | PC 2 |  | 2.4 | -0.023 | 0.01 | 2.3×10^-3^ |
| Android to Gynoid Ratio | Age | 6.3 | 3.8 | 0.033 | 0.01 | 2.3×10^-4^ |
|  | Sex, Male | 10.1 | 16.3 | 0.137 | 0.02 | 3.5×10^-13^ |
|  | HEI | 7.6 |  |  |  |  |
|  | PRS | 5.4 | 3.3 | 0.031 | 0.01 | 5.2×10^-4^ |
|  | RMR | 9.0 | 6.0 | -0.044 | 0.01 | 3.8×10^-6^ |
|  | Fitness | 9.4 | 10.8 |  |  |  |
|  |  |  |  | -0.137 | 0.04 | 5.9×10^-4^ |
|  |  |  |  | -0.080 | 0.04 | 0.022 |
|  |  |  |  | -0.012 | 0.04 | 0.75 |
|  |  |  |  | -0.033 | 0.04 | 0.35 |
|  |  |  |  | 0.016 | 0.03 | 0.63 |
|  |  |  |  | 0.058 | 0.03 | 0.71 |
|  |  |  |  | 0.031 | 0.04 | 0.47 |
|  | Combined PCs | 2.6 |  |  |  |  |
| Waist Circumference, log scale | Age | 1.9 |  |  |  |  |
|  | Sex, Male | 4.0 | 7.0 | 0.080 | 0.02 | 7.7×10^-7^ |
|  | HEI | 8.6 |  |  |  |  |
|  | PRS | 10.1 | 10.1 | 0.048 | 0.01 | 4.7×10^-9^ |
|  | RMR | 21.1 | 27.2 | -0.079 | 0.01 | 1.5×10^-19^ |
|  | Fitness | 5.5 |  |  |  |  |
|  | Combined PCs | 1.7 |  |  |  |  |
| Fasting Triglycerides, log scale | Age | 2.0 | 3.4 | 0.082 | 0.03 | 6.5×10^-3^ |
|  | Sex, Male | 0.6 |  |  |  |  |
|  | HEI | 5.9 | 5.7 | -0.107 | 0.03 | 4.0×10^-4^ |
|  | PRS | 2.2 |  |  |  |  |
|  | RMR | 0.2 |  |  |  |  |
|  | Fitness | 10.3 |  |  |  |  |
|  | Combined PCs | 0.7 |  |  |  |  |
| Fasting HDLc, log scale | Age | 0.3 |  |  |  |  |
|  | Sex, Male | 11.8 | 12.4 | -0.201 | 0.04 | 3.1×10^-8^ |
|  | HEI | 2.4 |  |  |  |  |
|  | PRS | 0.7 |  |  |  |  |
|  | RMR | 8.0 | 10.6 | 0.098 | 0.02 | 2.7×10^-7^ |
|  | Fitness | 6.3 |  |  |  |  |
|  | Combined PCs | 2.1 | 2.4 |  |  |  |
|  | PC 1 |  |  | 0.047 | 0.2 | 0.012 |
| Fasting Glucose | Age | 6.9 | 8.5 | 0.152 | 0.05 | 8.2×10^-6^ |
|  | Sex, Male | 2.9 | 3.6 | 0.194 | 0.03 | 3.2×10^-3^ |
|  | HEI | 4.1 | 3.0 | -0.091 | 0.07 | 7.5×10^-3^ |
|  | PRS | 2.4 | 2.4 | 0.0811 | 0.03 | 0.015 |
|  | RMR | 1.3 |  |  |  |  |
|  | Fitness | 4.5 |  |  |  |  |
|  | Combined PCs | 1.9 |  |  |  |  |
| Systolic Blood Pressure | Age | 3.6 | 4.0 | 2.09 | 0.70 | 3.2×10^-3^ |
|  | Sex, Male | 0.5 |  |  |  |  |
|  | HEI | 0.3 |  |  |  |  |
|  | PRS | 1.3 |  |  |  |  |
|  | RMR | 0.1 |  |  |  |  |
|  | Fitness | 8.8 |  |  |  |  |
|  | Combined PCs | 5.6 |  |  |  |  |
|  | PC 4 |  | 2.7 | -1.71 | 0.70 | 0.015 |
| Diastolic Blood Pressure | Age | 1.8 |  |  |  |  |
|  | Sex, Male | 4.3 | 5.1 | 4.02 | 1.19 | 8.6×10^-4^ |
|  | HEI | 0.3 |  |  |  |  |
|  | PRS | 0.4 |  |  |  |  |
|  | RMR | 2.1 | 3.0 | -1.56 | 0.60 | 9.4×10^-3^ |
|  | Fitness | 8.6 |  |  |  |  |
|  | Combined PCs | 1.7 |  |  |  |  |

The following variables were included in both analyses: age, sex, diet quality (HEI score), polygenic risk score (PRS), resting metabolic rate (RMR), fitness (YMCA Step Test), and principal components 1–5 (Combined PCs). The predictor variables were standardized for all models. The variance explained by predictor variables using sum of squares (SS) and partial R2 is listed. SS illustrates the proportion of variance in outcomes explained by each predictor variable, based on sum of squares from an ANOVA analysis. The total sum of squares is partitioned to show the relative contribution of each predictor to the overall variation in each outcome without accounting for the influence of other variables. Only variables identified in the best-fit model from the stepwise, bidirectional model building using Bayesian information criteria (BIC) are included under partial R2 and regression results. Partial R² quantifies the proportion of variance in the outcome explained by a predictor variable, after controlling for the effects of other predictors in the model. It reflects the unique contribution of each predictor to the outcome.


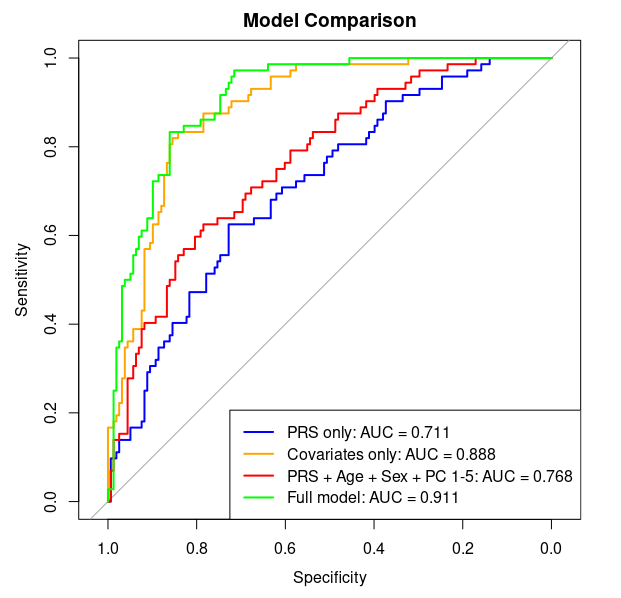


**Supplementary Figure 4**. Multiple ROC comparing predictive models including a PRS only, covariates only, PRS, age, sex, and PCs, and a full model including all predictor variables. The correlated ROC can be statistically compared using DeLong’s test. All compared models are significantly difference from one another. (ROCsimple vs ROCprs, p = 0.021; ROCprs vs ROCcovariates, p = 1.9e-05; ROCsimple vs ROCfull, p = 4.2e-06; ROCprs vs ROCfull, p = 1.1e-08; ROCcov vs ROCfull, p = 0.030)
